# Coronary Sinus Electrogram Changes After Cavotricuspid Isthmus Ablation for Typical Atrial Flutter: What Is the Mechanism?

**DOI:** 10.1002/joa3.70475

**Published:** 2026-10-03

**Authors:** Shun Nakamura, Takashi Nakashima, Takahisa Ido, Shigekiyo Takahashi, Takuma Aoyama

**Affiliations:** ^1^ Department of Cardiology Central Japan International Medical Center Gifu Japan; ^2^ Department of Cardiology, Graduate School of Medicine Gifu University Gifu Japan; ^3^ Department of Molecular Pathophysiology Shinshu University Graduate School of Medicine Nagano Japan

**Keywords:** atrial flutter, catheter ablation, cavotricuspid isthmus, coronary sinus, epicardial connection

## Abstract

Typical atrial flutter was terminated and sinus rhythm was restored. We discussed the mechanism of changes in the coronary sinus atrial electrograms.

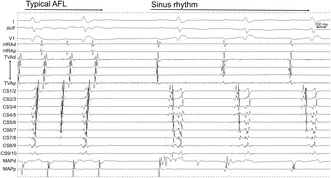

## 1 Case Presentation

A 60‐year‐old female with systemic scleroderma and interstitial pneumonia presented with a 2‐month history of persistent dyspnea and atrial flutter (AFL). Echocardiography showed a reduced left ventricular ejection fraction of 39% with mild mitral and tricuspid regurgitation.

The baseline rhythm was AFL with an atrial tachycardia‐cycle length of 256 ms. The catheter positions are shown in Figure 1A. Entrainment mapping and three‐dimensional electroanatomical mapping (CARTO, Biosense Webster Inc., Diamond Bar, CA, USA) confirmed typical counterclockwise AFL (Figure 1B,C). Following cavotricuspid isthmus (CTI) linear ablation, the AFL terminated and sinus rhythm was restored (Figure 2). Bidirectional CTI block was achieved. What is the mechanism of the changes in the atrial electrograms (EGMs) recorded in the coronary sinus (CS)?

**FIGURE 1:**
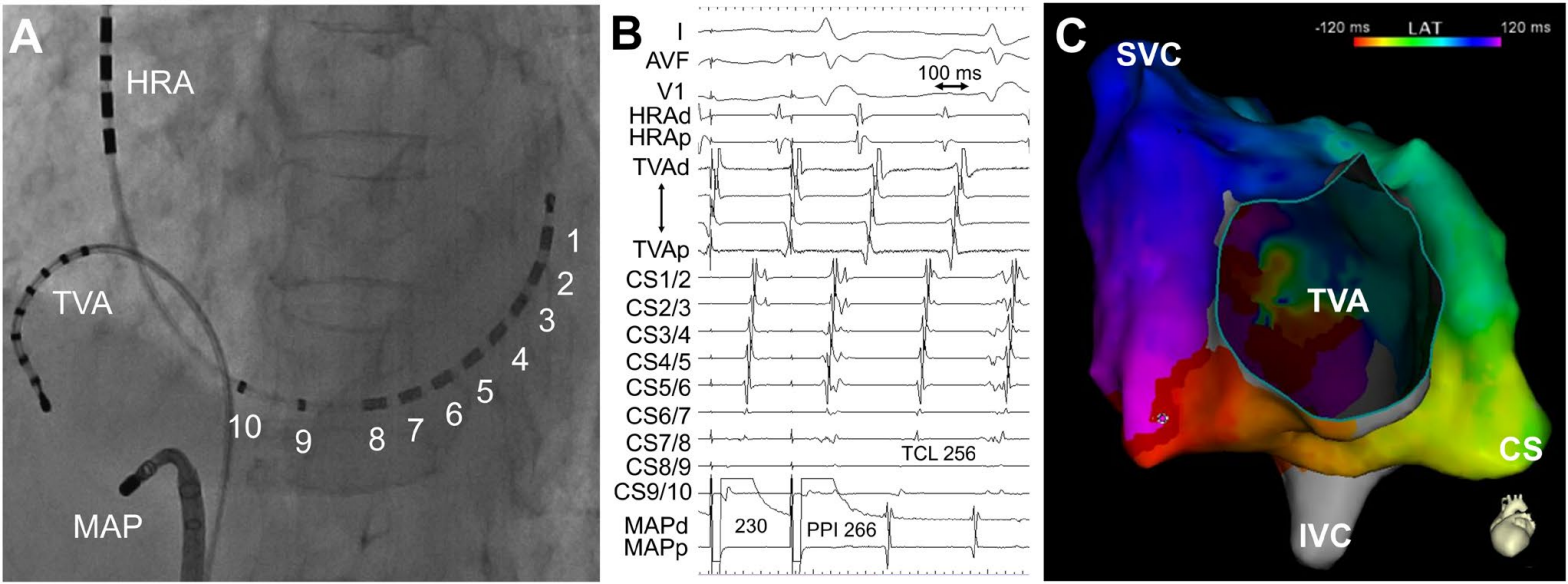
Electrophysiological findings during atrial flutter (AFL). (A) Fluoroscopic left anterior oblique view of catheter positions. The mapping catheter (MAP) is at the 7 o'clock position of the tricuspid valve annulus (TVA). (B) Entrainment mapping from the MAP catheter. The post‐pacing interval (PPI) equals the tachycardia cycle length (TCL), confirming that the catheter is within the reentry circuit. Values are expressed in milliseconds. (C) Three‐dimensional activation map demonstrating typical counterclockwise AFL. CS, coronary sinus; d, distal; HRA, high right atrium; IVC, inferior vena cava; p, proxima; SVC, superior vena cava.

**FIGURE 2:**
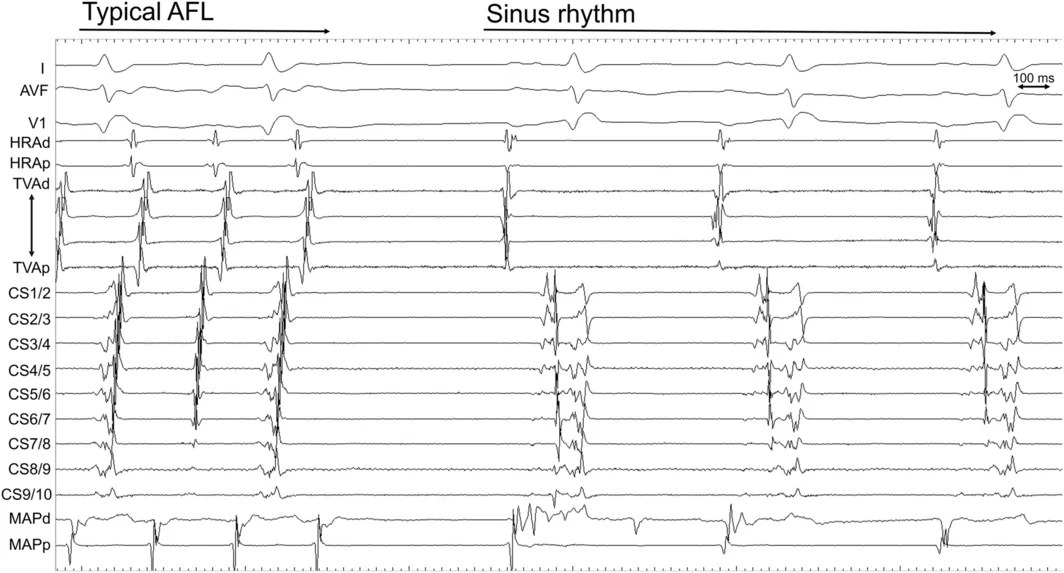
Intracardiac electrograms (EGMs) showing restoration of sinus rhythm following cavotricuspid isthmus (CTI) ablation. Abbreviations as in Figure 1.

## 2 Discussion

The present case demonstrates an epicardial connection between the low right atrium (RA) and the CS musculature, as elucidated by detailed EGM analysis (Figure 3). During sinus rhythm post‐AFL termination, double potentials were recorded along the CS electrodes. The first component, characterized by dull, low‐amplitude signals, exhibited a proximal‐to‐distal activation sequence, representing far‐field endocardial left atrial (LA) potentials (arrowheads in Figure 3A). The second component consisted of sharp, high‐amplitude signals with a distal‐to‐proximal activation sequence, representing near‐field epicardial CS musculature potential (open arrowheads in Figure 3A). These two EGMs merged in the distal CS, suggesting an endocardial LA and CS musculature connection distally, but not proximally. However, these two components were superimposed during AFL (arrow in Figure 3B). During entrainment pacing from mid‐CS 7/8, the double potentials separated, and their activation sequence was reversed from that during sinus rhythm. The first component was near‐field epicardial CS musculature potential with a proximal‐to‐distal sequence (arrowheads in Figure 3C), while the second was far‐field endocardial LA potentials with a distal‐to‐proximal sequence (open arrowheads in Figure 3C).

**FIGURE 3:**
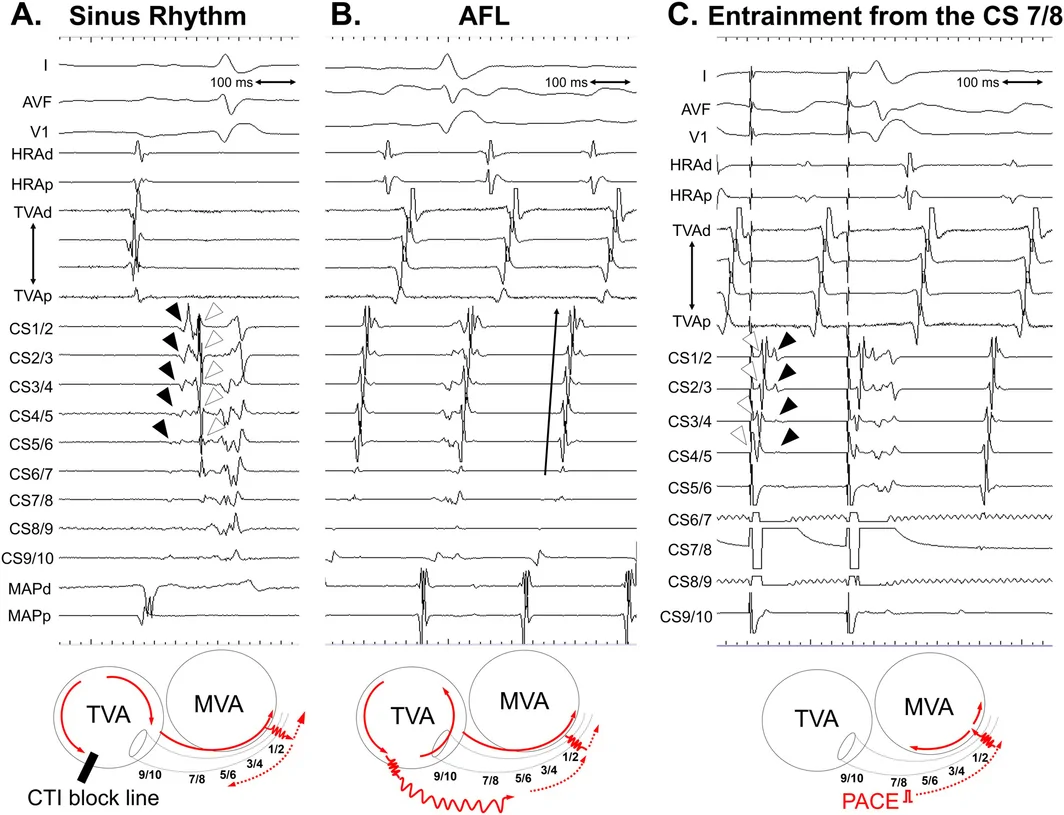
Mechanistic insights into CS EGMs changes. (A) Intracardiac EGMs during sinus rhythm. (B) Intracardiac EGMs during AFL. (C) Entrainment mapping from CS 7/8. Abbreviations as in Figures 1 and 2.

Another critical mechanistic insight was obtained when premature atrial contractions (PACs) were mechanically induced in the RA by the OCTARAY mapping catheter during AFL (Figure 4). During these PACs, the double potentials became separated, with the near‐field CS musculature EGM (open arrowheads in Figure 4) preceding the far‐field LA EGM (arrowheads in Figure 4). These observations confirm that the PACs conducted directly from the RA to the CS musculature via an epicardial bridge. Given that the double‐potential intervals and the activation sequence of the far‐field LA EGMs varied during mechanical PACs, multiple alternative pathways likely existed between the CS musculature and the LA.

**FIGURE 4:**
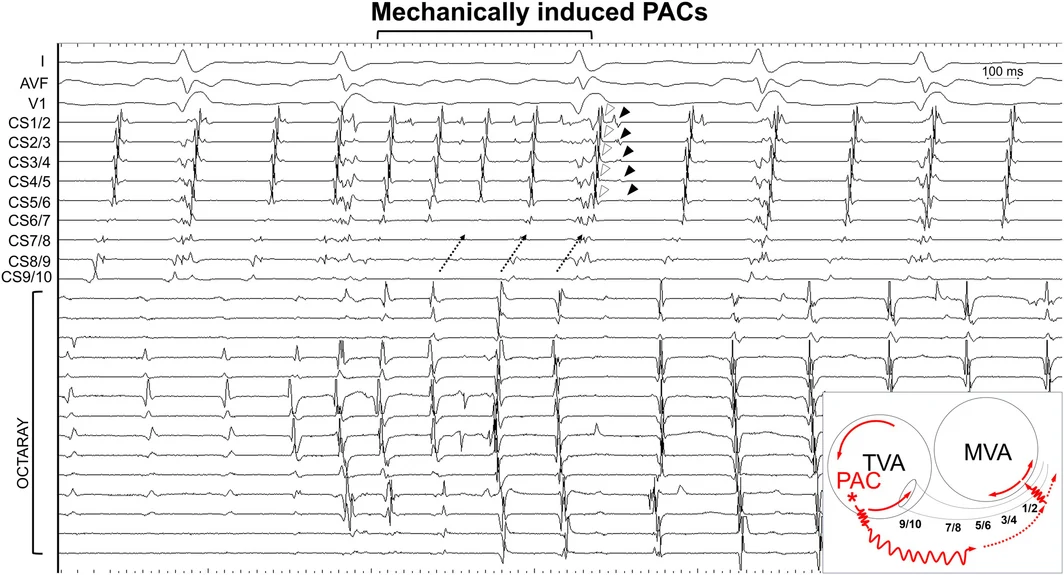
Intracardiac EGMs of mechanical PACs induced by the OCTARAY mapping catheter during AFL. Abbreviations as in Figures 1 and 2.

Based on these findings, we concluded that the LA was connected to the CS musculature at the distal CS, and that an epicardial connection existed between the RA and the CS musculature. CTI ablation eliminated this low RA‐to‐CS musculature connection. Several limitations should be noted in this case. First, because the baseline rhythm was AFL, CS EGMs could not be recorded during sinus rhythm prior to ablation. Second, although endocardial bidirectional block across the CTI line was confirmed, whether the epicardial connection between the RA and the CS musculature was blocked remained unverified. Third, the precise insertion site (epicardial‐to‐endocardial or endocardial‐to‐epicardial) was not fully clarified. Nonetheless, we believe that these limitations do not undermine the validity of our interpretation.

While RA‐to‐CS epicardial connections have been documented in animal models, clinical electrophysiological observations in humans remain rare [1].

Furthermore, reports describing this connection through the analysis of intracardiac EGMs within the CS have seldom been reported. Our case is noteworthy because the presence of a low RA‐to‐CS epicardial bridge was clearly demonstrated by evaluating EGM morphologies and activation patterns during both sinus rhythm and typical AFL.

## Funding

The authors have nothing to report.

## Ethics Statement

This study was approved by the institutional review board of Central Japan International Medical Center. The study complied with the principles of the Declaration of Helsinki.

## Consent

Informed consent was obtained from the patient.

## Conflicts of Interest

The authors declare no conflicts of interest.

## Data Availability

The data that support the findings of this study are available from the corresponding author upon reasonable request.
